# Evaluating the test re-test reliability and inter-subject variability of health care provider manual fluid resuscitation performance

**DOI:** 10.1186/1756-0500-7-724

**Published:** 2014-10-15

**Authors:** Melissa J Parker, Frank MH Lee, Lawrence Mbuagbaw, Lehana Thabane

**Affiliations:** Department of Pediatrics, McMaster Children’s Hospital and McMaster University, 1280 Main St W. Room 3A, Hamilton, Ontario L8S 4K1 Canada; Division of Emergency Medicine, Department of Paediatrics, The Hospital for Sick Children, and University of Toronto, 555 University Avenue, Toronto, Ontario M5G 1X8 Canada; Department of Clinical Epidemiology and Biostatistics, McMaster University, 1200 Main St W, Hamilton, Ontario L8N 3Z5 Canada; Biostatistics Unit,/FSORC, St Joseph’s Healthcare Hamilton, 3rd floor Martha Wing 50 Charlton Avenue East, Hamilton, L8N 4A6 Canada; Department of Anesthesia, McMaster University, 1280 Main St W, Hamilton, Ontario L8S 4K1 Canada; Division of Critical Care, Department of Pediatrics, McMaster Children’s Hospital and McMaster University, 1280 Main St. W, Room 3Y, Hamilton, Ontario L8S 4K1 Canada

**Keywords:** Research methods, Resuscitation, Shock, Fluid therapy, Pediatrics

## Abstract

**Background:**

Health Care Providers (HCPs) report that manual techniques of intravascular fluid resuscitation are commonly used during pediatric shock management. The optimal pediatric fluid resuscitation technique is currently unknown. We sought to determine HCP test-retest reliability (repeatability) and inter-subject variability of fluid resuscitation performance outcomes to inform the design of future studies.

**Methods:**

Fifteen consenting HCPs from McMaster Children’s Hospital, in Hamilton, Canada participated in this single-arm interventional trial. Participants were oriented to a non-clinical model representing a 15 kg toddler, which incorporated a 22-gauge IV catheter. Following a standardization procedure, participants administered 600 mL (40 mL/kg) of saline to the simulated child under emergency conditions using prefilled 60-mL syringes. Each participant completed 5 testing trials. All testing was video recorded, with fluid administration time outcome data (in seconds) extracted from trial videos by two blinded outcome assessors. Data describing catheter dislodgement events, volume of saline effectively delivered, and participant demographics were also collected. The primary outcome of fluid administration time test-retest reliability was analyzed by one-way analysis of variance (ANOVA) and intra-class correlation (ICC), with good reliability defined as ICC > 0.70.

**Results:**

Differences in HCP fluid administration times are attributable to inter-subject variability rather than intra-subject variability based on one-way ANOVA analysis, F (14,60) = 43.125; p < 0.001. Test-retest reliability of subjects was excellent with ICC = 0.97 (95% CI: 0.95-0.99); p < 0.001.

**Conclusions:**

Findings demonstrate excellent test-retest reliability of HCP fluid resuscitation performance in a setting involving a non-clinical model. Investigators can justify a single evaluation of HCP performance in future studies.

**Electronic supplementary material:**

The online version of this article (doi:10.1186/1756-0500-7-724) contains supplementary material, which is available to authorized users.

## Background

Shock is a frequently encountered pediatric medical emergency. Current guidelines from the European Resuscitation Council, the American Heart Association, and the American College of Critical Care Medicine recommend prompt and rapid intravascular volume administration for the treatment of shock [1–3].

Prior research has demonstrated that children experiencing shock frequently do not receive timely fluid resuscitation according to current guidelines recommendations, and that this is linked with decreased survival odds [4–7]. To improve adherence to fluid resuscitation guideline goals, clear recommendations regarding how this task is optimally performed are required. Currently, Health Care Providers (HCPs) use a variety of techniques to accomplish rapid intravascular volume administration for children in shock [8] without any one technique recommended or demonstrated as clearly superior. A lack of evidence regarding the efficiency of different fluid resuscitation methods is currently a barrier to development of clear recommendations.

Further research evaluating the efficiency of different fluid resuscitation techniques as performed by typical health care providers will help to inform future pediatric resuscitation guidelines and improve knowledge translation. While it is possible to conduct such research in the in vivo setting, studies evaluating health care provider performance in a setting utilizing a simulated patient may be equally informative and more cost-effective to conduct. For research planning purposes, an understanding of outcome data reliability is important. The objective of this study was therefore to evaluate HCP test-retest reliability (repeatability) and inter-subject variability of fluid resuscitation performance outcomes to inform the design of future studies. We hypothesized that good test-retest reliability would be demonstrated but that significant inter-subject variability would exist.

## Methods

### Study design

This study was a single-blind, non-clinical, interventional trial with one study arm. Approval for study conduct was obtained from the Faculty of Health Sciences/Hamilton Health Sciences Research Ethics Board (Project Number 12–125). Written informed consent was obtained from all participants prior to participation.

### Study setting and population

The trial was conducted at McMaster Children’s Hospital, a tertiary pediatric academic center in Hamilton, Canada. The study setting was non-clinical, but we enrolled and evaluated human participants. Eligible participants included HCPs at McMaster Children’s Hospital. We defined HCPs as staff physicians, staff nurses, postgraduate medical trainees, and medical students. Exclusion criteria included non-English speaking individuals and those incapable of performing manual fluid administration using syringes. The study aimed to recruit a convenience sample of 15 participants through email invitation and a poster campaign, with a $25 coffee card offered as a participation incentive.

### Pediatric fluid resuscitation model

**Figure 1:**
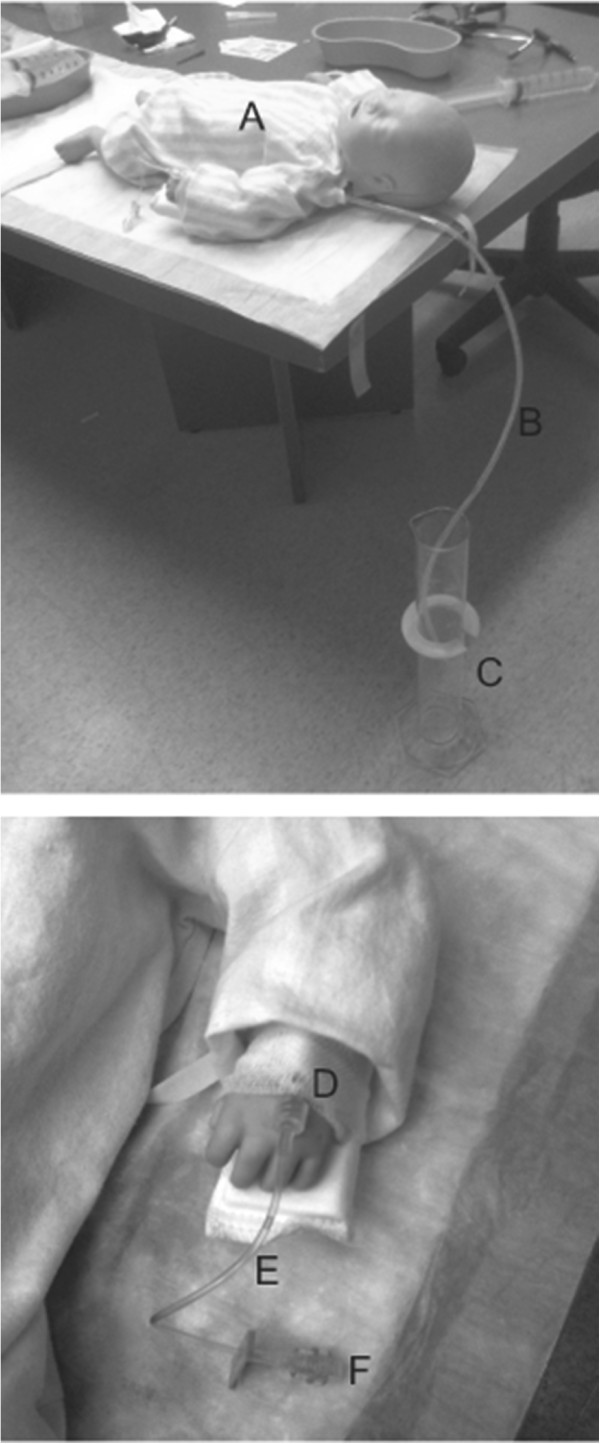
**Pediatric fluid resuscitation model. A**. Model Simulating 15 kg Child, **B**. Conduit Tubing, **C**. 1-Litre Graduated Cylinder, **D**. 1.00 inch, 22-gauge IV BD Insyte™ Autoguard IV catheter, **E**. Baxter 7.00 inch IV Catheter Extension Set, **F**. Baxter one-link needle-free IV connector.

The model used in this study (Figure 1) has been described in detail elsewhere [9]. Briefly, the model is a toddler-sized mannequin which incorporates a 1.00 inch, 22-gauge, BD Insyte™ Autoguard IV catheter to simulate in vivo conditions. The catheter is affixed to the hand of the model using waterproof tape, cling, and an arm board in typical clinical fashion. Fluid effectively administered to the model collects within a 1 litre graduated cylinder.

### Intervention

**Figure 2:**
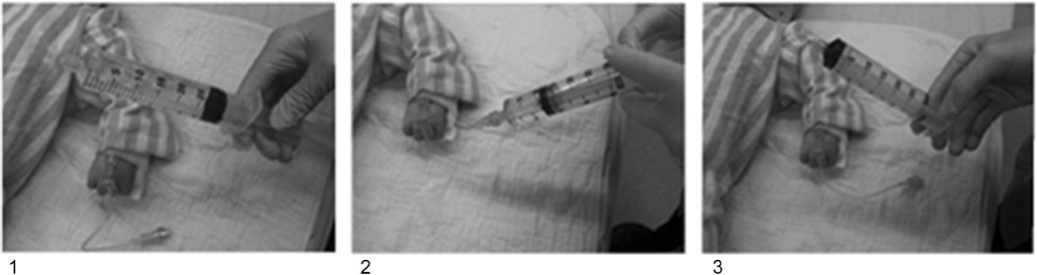
**The 'Disconnect-reconnect' technique of manual fluid resuscitation using syringes. 1**. A Health Care Provider takes a syringe filled with isotonic fluid prepared by a colleague, **2**. The Provider connects the syringe to the IV extension tubing, **3**. The Provider administers the isotonic fluid contained within the syringe to the patient by depressing the syringe plunger. Steps **1**–**3** are repeated as quickly as possible until the desired volume of fluid had been administered.

The study intervention consisted of the administration of 600 mL of 0.9% normal saline to the simulated child using 60 mL Luer-Lok™ syringes and the ‘disconnection-reconnection’ fluid resuscitation technique. The ‘disconnection-reconnection’ technique is illustrated in Figure 2, and has been described elsewhere [8]. We selected this technique for use in this study as it is a common and preferred form of manual fluid administration among pediatric heath care providers [8]. The protocol called for each participant to repeat the intervention 5 times to allow for calculation of study outcomes.

### Participant testing procedures

A research assistant was responsible for preparing the study setting and supplies. After obtaining written consent, each participant was oriented and underwent a standardization procedure to review and practice performing manual fluid administration to the model using the study method [10]. HCPs then underwent 5 testing trials, each separated by a washout period of a minimum of 10 minutes. Each trial was initiated on verbal prompt, with HCPs advised that the child was experiencing decompensated septic shock requiring IV fluid resuscitation as emergency treatment.

### Data collection and outcome ascertainment

All participant testing was video-recorded using a high definition video camera with a timing function. Filming was limited to the location of the IV catheter site. In addition to video recording of each trial, the research assistant recorded catheter dislodgement events, the volume of fluid effectively administered to the model as determined by the amount of fluid collected in the graduated cylinder, and the presence or absence of any testing irregularities. After completing all 5 trials participants answered a brief questionnaire to provide demographic information.

Two blinded outcome assessors independently viewed and extracted fluid administration time data from the trial video-recordings using a previously employed standard procedure [11]. The outcome assessors only had access to the study ID number associated with each video-recording and were not aware of any other data collected. Trial intervention time was determined by averaging the times recorded by Assessor 1 and Assessor 2 for each trial. All extracted data was used to calculate the inter-rater reliability of the assessors.

### Data management and statistical analysis

The raw data of interest was recorded on a data collection form, and then entered into a secure SPSS Version 20 database (IBM Corporation, Armonk, NY, USA). Our statistical analysis plan included analysis of participant demographic characteristics and outcome variables (both primary and secondary) with these summarized using descriptive summary measures. We also planned to use analysis of variance (ANOVA) and intra-class correlation (ICC) to determine HCP test re-test reliability, with good reliability defined as ICC > 0.70 [12]. ANOVA was used to assess the degree of inter-subject variability of intervention times. The ICC was also used to assess the inter-rater reliability of the two blinded outcome assessors.

## Results

**Figure 3:**
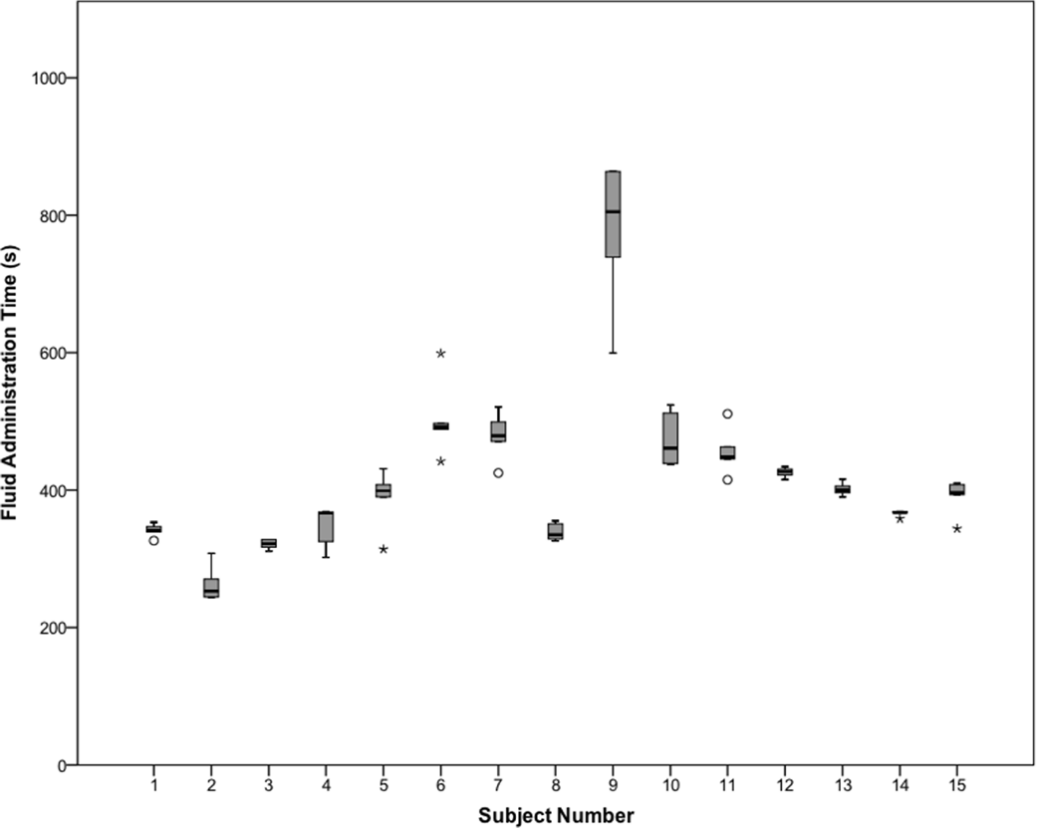
**Box-whisker plot of health care provider fluid administration time outcome data.** The box-whisker plot provides a visual display of the distribution of outcome data for each participant. The boxes depict the interquartile range, with the solid horizontal line within the box representing the median. The whiskers illustrate the distribution of data outside the interquartile range. Circles denote outliers; asterisks denote extreme outliers.

We recruited 15 participants from September 2012 to February 2013 with no potential participants excluded. (Table 1) HCP fluid administration time data is displayed in boxplot form in Figure 3, while a descriptive summary of the data is available online (see Additional file 1: Table S1, descriptive data). Differences in HCP fluid administration times are attributable to inter-subject variability rather than intra-subject variability based on one-way ANOVA analysis, F (14,60) = 43.125; p < 0.001. Test-retest reliability of subjects was excellent with ICC = 0.97 (95% CI: 0.95-0.99); p < 0.001. Variation in fluid administration time was unrelated to progression from trial 1 to 5, confirming that HCP performance did not reduce or improve with each successive attempt, F (4,70) = 0.54; p = 0.906. Correlation between trial number and fluid administration time was also low (r = 0.1; n = 75; p = 0.395). Agreement between the 2 independent and blinded outcome assessors over the 75 measurements (15 participants x 5 trials) was excellent, with ICC >0.97; p < 0.001 and a perfect Pearson’s correlation of r = 1; n = 75; p < 0.001. Differences in the saline volume effectively delivered to the model were also attributable to inter-subject rather than intra-subject performance variability based on one-way ANOVA analysis, F (14,60) = 3.867; p < 0.001. A single catheter dislodgement episode occurred in the 75 trials conducted (1.3% of trials).

**Table 1:** **Participant demographic data**

Variable	Number of participants
	N (%)
Age Range	
20–29 years of age	8 (53)
30–39 years of age	4 (27)
40–49 years of age	3 (20)
Male Gender	
No	9 (60)
Yes	6 (40)
Profession	
Medical Student	3 (20)
Postgraduate Trainee (Resident or Fellow)	4 (27)
Staff Physician	2 (13)
Staff Nurse	6 (40)
Full Time status	
No	1 (7)
Yes	14 (93)
Years Since M.D. or R.N. degree	
<5 years	7 (47)
5–9 years	3 (20)
10–19 years	3 (20)
20 years or more	2 (13)
Fluid Resuscitation Experience	
None	2 (13)
Minimal	4 (27)
Some	2 (13)
Experienced	3 (20)
Very Experienced	4 (27)

## Discussion

This study demonstrates excellent test-retest reliability (repeatability) of HCP fluid resuscitation performance in a setting involving a simulated patient. While visual inspection of the test-retest data (Figure 3) may suggest differences in performance reliability between individuals this was not borne out in our analyses. Our findings are useful from a research planning perspective, as the data support evaluating provider performance only once for each intervention under study.

In the clinical compared to the simulated setting, it is possible that patient movement could impact test-retest reliability. This consideration is not relevant to moribund patients, sedated and mechanically ventilated patients, or patients in the operating room under general anesthesia. Patients with decompensated shock in whom manual fluid administration techniques would be used would be expected to move very little if at all. Therefore our findings may also be applicable to these and similar clinical contexts.

We had speculated that within subject differences in performance might exist and could be caused by either a learning effect or provider fatigability. To control for a potential learning effect, we employed a standardization procedure. The possibility of provider fatigability certainly was a concern due to findings from our previous work in which HCPs reported increasing fatigue with performance of manual fluid resuscitation using syringes [11]. For this reason, we designed our protocol to include a washout period between each trial. Our finding of no association between number of trials and HCP fluid administration times supports the conclusion that a training effect was not observed, and that the washout period used was adequate to prevent any potential impact of fatigue on provider performance.

We did observe significant differences in performance between HCPs consistent with our hypothesis. The reason(s) for inter-individual differences are unclear but could relate to strength, dexterity, experience, confidence in performing the required task, or other factors. We attempted to mitigate the potential impact of experience by including a standardization procedure in our protocol and giving participants an opportunity to practice prior to formal testing. Our sample size is not sufficiently large to assess the potential impact of demographic factors on performance. However our findings do support the use of a randomized controlled trial design in any future comparative studies to control for known and unknown confounders.

The finding of a high level of agreement between the blinded outcome assessors extracting performance outcome data from video recordings using a standardized procedure affirms our previous findings [11]. While this result specifically relates to extraction of fluid administration time outcome data, it is perhaps of interest and relevant to other researchers interested in extracting resuscitation outcome data from video-recordings.

There are several limitations in our study. Because this study was conducted in a non-clinical setting involving a simulated patient, our results may differ from research in a clinical setting involving human subjects. For our objectives this is acceptable since study results are intended to inform research under similar conditions. Secondly, when evaluating test-retest reliability and inter-subject variability it is ideal from a statistical perspective to have a very large sample of subjects and to test their performance as many times as possible. Given our needs and feasibility considerations, we determined that recruiting 15 subjects and having them perform the intervention 5 times would provide sufficient data to answer our research question.

## Conclusions

The findings of this study demonstrate excellent test-retest reliability of HCP fluid resuscitation performance in a setting involving a simulated patient. Future research evaluating HCP fluid resuscitation performance can justify evaluating HCPs on a single occasion for each intervention of interest.

## Authors’ information

MP is an Assistant Professor of Pediatrics at McMaster University. She practices Pediatric Critical Care and Pediatric Emergency Medicine, and her area of research focus is pediatric resuscitation science. She is supported by a Hamilton Sciences Research Early Career Award.

FL is currently an MD/PhD student at the University of British Columbia. He conducted this work while an undergraduate student in the Bachelor of Health Sciences Program at McMaster University.

LM is a PhD student in the Department of Clinical Epidemiology and Biostatistics at McMaster University.

LT is a Professor and Associate Chair, Department of Clinical Epidemiology & Biostatistics, and Director of the Centre for Evaluation of Medicine.

## Electronic supplementary material

Additional file 1: Table S1: Fluid Administration Time Outcome Data and Descriptive Summary Measures. (PDF 32 KB)

## Abbreviations

ACCM: American College of Critical Care Medicine
ERC: European Resuscitation Council
HCP: Health care provider
PALS: Pediatric Advanced Life Support
ANOVA: Analysis of variance
ICC: Intra-class correlation.
