# Supplementary material for: Evaluating the test re-test reliability and inter-subject variability of health care provider manual fluid resuscitation performance
Source: BMC Res Notes. 2014 Oct 15;7:724. doi: 10.1186/1756-0500-7-724 (PMC4210565; doi:10.1186/1756-0500-7-724)
Supplement: Supplementary file 1 — Additional file 1: Table S1: Fluid Administration Time Outcome Data and Descriptive Summary Measures. (PDF 32 KB) [file 13104_2014_3254_MOESM1_ESM.pdf]

Supplemental Digital Content 1. Fluid Administration Time Outcome Data and Descriptive Summary Measures

Subject Number	N	Mean (seconds)	Median (seconds)	Standard Deviation	Minimum (seconds)	Maximum (seconds)
1	5	341.4	341.0	9.89571	326.5	353.0
2	5	264.0	253.0	26.83049	244.0	308.0
3	5	321.2	322.0	7.32803	311.0	328.0
4	5	346.0	366.0	30.78149	302.0	369.0
5	5	388.4	399.0	44.29786	314.0	431.0
6	5	503.7	492.0	57.66671	442.0	599.0
7	5	479.0	479.0	35.86084	425.0	521.0
8	5	339.2	335.0	13.08434	326.0	355.0
9	5	774.2	805.0	110.39282	599.5	864.0
10	5	474.6	461.0	40.94264	437.0	524.0
11	5	456.5	448.0	35.09986	415.0	511.0
12	5	425.8	427.0	7.26808	415.5	434.0
13	5	401.6	400.0	9.93982	390.0	416.0
14	5	366.0	367.0	4.58258	358.0	369.0
15	5	390.3	396.0	26.86913	344.0	410.0
Total	75	418.1	399.0	121.17017	244.0	864.0
